# Randomized Controlled Trials in Environmental Health Research: Unethical or Underutilized?

**DOI:** 10.1371/journal.pmed.1001775

**Published:** 2015-01-06

**Authors:** Ryan W. Allen, Prabjit K. Barn, Bruce P. Lanphear

**Affiliations:** 1 Faculty of Health Sciences, Simon Fraser University, Burnaby, British Columbia, Canada; 2 Child and Family Research Institute, BC Children's Hospital, Vancouver, British Columbia, Canada

## Abstract

Ryan Allen and colleagues argue that more randomized controlled trials in environmental health would complement a strong tradition of observational research.

*Please see later in the article for the Editors' Summary*

**Summary Points**

- Efficacious environmental interventions are needed because environmental risks account for a large fraction of the global disease burden.
- Randomized controlled trials have not been widely embraced by environmental health researchers and comprise less than 1% of research publications in the field.
- Additional randomized controlled trials in environmental health would complement a strong tradition of observational research by creating new knowledge on exposure–health relationships, providing more definitive evidence of causality, identifying efficacious interventions to reduce or eliminate hazards, and countering the perception that environmental risks are evaluated with inadequate rigor.
- Ethical issues—including clinical equipoise, the distribution of benefits and risks, and the relevance of the intervention and health outcome to the study population—must be carefully considered before conducting a randomized controlled trial of an environmental intervention.

Randomized controlled trials (RCTs) are considered the “gold standard” study design in health research. The random allocation of participants to intervention and control groups minimizes systematic differences between groups and the biases that can result. RCTs have become standard practice in the evaluation of medical and pharmaceutical treatments. In contrast, environmental (and occupational) health research has relied primarily on observational methods; randomized studies to test the effect of an environmental exposure or the efficacy of an intervention to prevent or reduce exposure are rare.

A search of PubMed articles in journals focused on medicine, environmental health, and clinical trials revealed that only 0.6% of environmental health publications since 2000 were RCTs of an intervention to reduce exposure (S1 Text). RCTs contribute a larger portion of the environmental health publications in top-ranked medical journals (4%) than in environmental health journals (0.4%)—an unsurprising result given the emphasis on clinical trials in medical research and the widespread perception that observational studies are inferior. The RCTs published to date have focused primarily on allergens, drinking water, household air pollution (HAP) from solid cooking fuels, lead, environmental tobacco smoke, and pesticides.

RCTs should be used more frequently to study environmental hazards (see Box 1). In calling for more randomized studies of interventions our objective is not to offer yet another admonishment of observational epidemiology [1]–[3]. The contributions that observational research has made to our understanding of environmental risks and the development of environmental health policy are impressive. Notable examples include ambient air pollution [4], lead [5], radon [6], arsenic [7], and asbestos [8], all of which are now known to cause substantial morbidity and mortality, and have policies in place to mitigate their health risks, based almost entirely on observational evidence [9]. Well-designed and carefully conducted randomized trials would complement this strong tradition of observational research. The fundamental advantages of randomized designs, such as minimization of confounding bias, are described elsewhere and need not be reiterated here. Instead, we aim to highlight how RCTs might be beneficial to environmental health research and describe some considerations for the appropriate use of RCTs to assess environmental risks and the efficacy of interventions.

**Box 1. Randomized Controlled Trials**

The RCT is a powerful research design that may have applicability to a wide range of risk factors in the physical, built, and social environments. RCTs may be appropriate and should be considered when the following criteria are met: (1) there is uncertainty about which (if any) intervention is most effective (i.e., clinical equipoise) or the gold standard intervention is not being implemented; (2) the intervention to be studied is feasible and affordable to the local community; (3) the intervention addresses a health risk affecting the local community; and (4) the health outcome and the timescale of the exposure–response relationship can feasibly be studied using the RCT design.

## Why Are More RCTs Needed?

Interventions to reduce or eliminate environmental exposures are urgently needed; environmental risks account for 13%–37% of the disease burden (quantified by disability-adjusted life years) in individual countries [10],[11]. The individual-level health risks of environmental exposures are often modest, but the population-level impacts are substantial because exposures are highly prevalent or ubiquitous and contribute to common diseases and disabilities [9]. Environmental exposures affect health in both high-income countries and low- and middle-income countries (LMIC), although the relative importance of specific risk factors and the magnitude of the risks vary with economic development [12].

RCTs can generally provide more definitive evidence of causality than observational studies. As a result, greater use of RCTs in environmental health would help to emphasize prevention over treatment by altering the perception that environmental risks are evaluated less rigorously than medical and pharmaceutical interventions. As previously noted in the context of HAP, the perception that environmental interventions are evaluated with insufficient rigor has important implications for the allocation of limited resources:

“[Randomized] studies would go a long way in making the argument about causality to health ministries and international agencies that support them, who usually have very limited resources to deal with a number of large health problems. They have the results of rigorous studies focused on other means of dealing with these same diseases… At present, as the effectiveness and cost of such measures has been much better established, [HAP] interventions garner little attraction. On the other hand, the currently available interventions are clearly imperfect and will not serve to entirely control the diseases” [13].

Environmental interventions are cost-effective investments [14]. For example, each dollar invested to control lead hazards in the United States would result in benefits of $17–$221 [15]. By 2020 the cumulative benefits of the 1990 US Clean Air Act Amendments are projected to outweigh costs by a ratio of 30 to 1 [16]. Globally, the annual economic benefit of halving the population without access to improved cooking stoves is estimated at $105 billion compared to a net cost of $34 billion [17].

Well-designed RCTs can also identify inefficacious or even harmful interventions. For example, several studies reported that residential lead abatement increased children's blood lead levels, which led to post-abatement lead-dust standards to protect children from the short-term risks of lead released during abatement or renovation [18],[19]. Even if they do not increase health risks, non-efficacious interventions can be a waste of scarce resources and provide a false sense of safety [20],[21]. In the absence of evidence, individuals will often turn to unproven solutions—like surgical masks, which are commonplace in many cities with poor air quality but do little to reduce health risks from air pollution [22].

## Individuals Versus Populations

Most environmental health RCTs conducted to date have evaluated interventions on individuals or households, but RCTs may also be appropriate for evaluating interventions implemented at the community level. For example, Patil and colleagues conducted an RCT in 80 villages to evaluate the effect of India's Total Sanitation Campaign (TSC) on the availability of improved sanitation, open defecation behaviors, water quality, and childhood diarrheal and gastrointestinal illnesses [23]. Villages randomized to the intervention group received the TSC, while control group villages received the TSC after the trial was completed. In other situations, it may be infeasible (or unethical) to randomize communities to the intervention, and “natural experiments” evaluating temporal changes in exposure and health may be a more appropriate design [24]–[27].

From a public health perspective, programs that reduce pollution emissions and exposure among large populations will always be preferable to interventions that attempt to reduce exposure at the individual or household level after the pollutants have been widely distributed. Nevertheless, RCT evaluations of interventions at the individual or household level can have tremendous value. Robust evidence that exposure reductions lead to improvements in health—even at the individual level—may make the argument for policy interventions at the population level more persuasive. The simplicity of the RCT design also makes it relatively easy to communicate results to non-researchers. Thus, even if the intervention under study is not feasible on a large scale, the information generated on exposure–health relationships may have relevance to population health and policy. Using RCTs to demonstrate efficacy for individual- or household-level interventions can also be valuable if emissions or production cannot be directly controlled. For example, wildfire smoke and radon are naturally occurring pollutants for which risk management involves exposure reduction primarily at the household level [28],[29].

RCTs are ideal for demonstrating efficacy (performance of the intervention under optimal conditions) but are generally inadequate for evaluating effectiveness (performance of the intervention under “real world” conditions) [30]. In clinical trials external validity is often limited because the demographics and health of study populations differ from their target populations [31]. External validity for RCTs in environmental health can be further influenced by the complexity of “scaling-up” interventions to a larger population [32]. An intervention's impact on health at the population level depends on efficacy, but also on user compliance, delivery, programming, and government policy [33]. Thus, RCTs generally represent the first step in developing an effective environmental intervention program.

Some have suggested that the relatively small study populations in RCTs are only capable of detecting “large” health effects and that RCTs are therefore not useful in public (or environmental) health research [34]. However, this is not necessarily the case because the small populations in RCTs are offset by three advantages. First, the smaller populations in RCTs often allow for refined, individual-level exposure assessment using environmental measurements and/or exposure biomarkers that is often not possible in larger observational studies. A reduction of non-differential exposure misclassification can dramatically enhance our ability to detect relationships with health outcomes. Second, the ability to detect relationships between exposure and health depends, in part, on the size of the exposure gradient in the population. Ironically, it is the ubiquity of many environmental exposures that makes their relationship with health difficult to uncover because “the hardest cause to identify is one that is universally present, for then it has no influence on the distribution of disease” [35]. RCTs can substantially increase the exposure gradient in the study population—and thus our ability to detect associations with health—by reducing exposure in the intervention group. Finally, environmental health researchers often struggle to identify the causative agent because many exposures share common sources and are therefore correlated. By reducing one specific exposure we can decrease its correlations with other exposures and more clearly identify the key agent(s) impacting health. For example, high efficiency particulate air (HEPA) filters reduce particulate matter (PM) air pollution concentrations indoors, but they have little effect on gaseous pollutants [36]. Thus, studies demonstrating health benefits from HEPA filtration provide evidence that PM plays an important role [36]–[38].

## Ethical Considerations

RCTs raise important ethical questions, and while a comprehensive review of ethical considerations is beyond the scope of this essay, some issues with relevance to environmental health research should be mentioned. The fundamental question with all RCTs is whether it is ethical to provide a potentially beneficial treatment or intervention to some participants but not to others [39]. It is widely accepted that RCTs are only ethical in situations of clinical equipoise—genuine uncertainty among the community of experts about which (if any) intervention is most effective [40]. No participant in a randomized trial should receive a placebo or an inferior intervention if an efficacious intervention has been identified [41]. It is often assumed that participants assigned to the intervention group in an RCT will benefit, but if equipoise exists the intervention is as likely to be non-efficacious as it is to be beneficial [20],[42], and in some cases the intervention may unintentionally increase exposure [19],[43].

Research participants should receive a fair share of the benefits from the research and the benefits to participants and society should be proportional to or outweigh the risks [39],[44]. In addition, the research must address a health problem of relevance to the population under study and the population should be selected based on the research objectives, not the population's vulnerability [45]. These considerations also have applicability to RCTs conducted in LMIC. While many clinical trials are now conducted in LMIC for financial reasons [46], many randomized studies of environmental interventions focus on LMIC because that is where the public health burden of many environmental risks is greatest and where the risks are often concentrated at the household level [9],[12]. These studies are ethical only if the intervention being tested is feasible and affordable in the local context and addresses a health risk of relevance to the population under study. Finally, researchers should clearly communicate that the study will not intentionally increase exposure to environmental hazards [47].

## Other Considerations

An important consideration for the RCT design is the timescale of the exposure–health relationship. It may be prohibitively expensive and difficult to mount RCTs for studying diseases with long latency periods, such as cancer or cardiovascular disease, but the RCT may be useful for testing the efficacy of interventions to reduce exposure to known carcinogens or cardiovascular risks (e.g. arsenic, lead) or for studying intermediate biological processes (e.g., systemic inflammation, endothelial dysfunction). The RCT design should also be considered in studies of acute or sub-chronic health effects and/or in situations with a well-defined exposure period of interest (e.g., pregnancy).

A key difference between clinical trials and RCTs of environmental interventions is that in the latter it is often difficult to blind participants and research personnel to intervention status. This may lead to biased effect estimates, particularly when investigating subjectively assessed health outcomes [48]. For example, unblinded studies of water treatment interventions in LMIC suggest that interventions produce substantial reductions in risk of diarrhea, while a smaller number of blinded studies have not found comparable benefits [49]. Even if participants cannot be blinded researchers should strive to rely on “hard,” objective outcomes, and personnel responsible for outcome assessment should be blinded (i.e., single blind).

Although many of the environmental health RCTs conducted to date have studied relatively simple interventions focused on a single environmental hazard, some have evaluated multifactorial interventions aimed at multiple exposures [50],[51]. Other fields have applied the RCT design more frequently and ambitiously. For example, the “Moving to Opportunities” study randomized over 4,000 families in high-poverty areas of several US cities into one of two housing mobility intervention groups or a control group and evaluated relationships with a range of outcomes [52]–[56]. The MIT Poverty Action Lab is using RCTs to address a wide range of questions in international development [57].

Our ongoing Ulaanbaatar Gestation and Air Pollution Research (UGAAR) study provides a useful example of a RCT in environmental health. Several large, well-conducted observational studies have suggested a link between ambient air pollution and impaired fetal growth [58],[59], but concerns about confounding and exposure misclassification remain [60]. We are randomizing approximately 500 pregnant women into either a HEPA filter intervention group or a control group (no filter); women in the intervention group will have HEPA filters operating in their home from enrollment until the child's birth. Ulaanbaatar, Mongolia is an ideal location to test the efficacy of this intervention because it has extraordinarily high air pollution concentrations [61] that are likely to remain elevated for decades even under the most optimistic scenarios [62],[63]. This situation is not unique—nearly 90% of the world's population breathes air pollution that exceeds WHO guidelines, and concentrations are increasing in much of the world [64] —so there is value in identifying efficacious interventions that can reduce health risks in the near term until regulations, technology, and economic development can reduce air pollution to acceptable levels. The randomized design should minimize confounding, and the high pollution concentrations and large exposure gradients created by the intervention provide statistical power among a relatively small study population on which detailed, household-level exposure assessment can be conducted. HEPA filters are a feasible and affordable intervention, and impaired fetal growth is a relevant outcome in this population.

## Conclusions

Randomized controlled trials are standard practice in clinical and pharmaceutical research but have not been embraced by environmental health researchers. Greater use of the RCT design would complement the tremendous contributions made by other methods—including both observational epidemiology and toxicology—to our understanding of environmental risks and the development of environmental health policy. Researchers, academic institutions, and funding agencies have a role to play in expanding the use of RCTs in environmental health research. Researchers should think creatively about potential interventions and consider the RCT as a possible study design to test their specific research question. Funding agencies should allocate money specifically for randomized studies of environmental interventions. In addition to its scientific advantages, this would provide the additional benefit of encouraging research that aims not only to identify problems but also to identify possible solutions. Ethical issues must be considered carefully, and while institutional ethics approval is necessary, it is not sufficient to ensure that the research is conducted ethically. The RCT design has important limitations and is not applicable to all research questions, so observational studies will, and should, remain the workhorse in environmental health research. Nevertheless, RCTs can help advance the field of environmental health by creating new knowledge of exposure–health relationships, providing more definitive evidence of causality, identifying efficacious interventions to reduce or eliminate exposure and health risks, and countering the perception that environmental risks are evaluated with inadequate rigor.

## Supporting Information

S1 Text **Details of PubMed search for randomized controlled trials in environmental health.**
(DOCX)

## Abbreviations

HAP: household air pollution
HEPA: high efficiency particulate air
LMIC: low- and middle-income countries
PM: particulate matter
RCT: randomized controlled trial
TSC: Total Sanitation Campaign
UGAAR: Ulaanbaatar Gestation and Air Pollution Research
