# Supplementary material for: Randomized Controlled Trials in Environmental Health Research: Unethical or Underutilized?
Source: PLoS Med. 2015 Jan 6;12(1):e1001775. doi: 10.1371/journal.pmed.1001775 (PMC4285396; doi:10.1371/journal.pmed.1001775)
Supplement: S1 Text — Details of PubMed search for randomized controlled trials in environmental health. (DOCX) [file pmed.1001775.s001.docx]

**Text S1:**

**Details of PubMed Search for Randomized Controlled Trials in Environmental Health**

We searched in papers published in key journals between January 1, 2000 and May 31, 2014 for RCTs using specific keywords intended to capture “mainstream” environmental health research topics. To ensure that our search terms captured the vast majority of environmental health publications, we first ran the search only on *Environmental Health Perspectives* and found that the search terms captured 85% of all papers published in that journal during the search period. We then expanded our search to include five journals focused on environmental health (*Environmental Health Perspectives*, *Environmental Research, Epidemiology*, *Journal of Exposure Science & Environmental Epidemiology*, and *Occupational and Environmental Medicine*), six top-tier medical journals (*BMJ, JAMA, New England Journal of Medicine*, *Lancet*, *Pediatrics*, and *PLOS Medicine*), and *Contemporary Clinical Trials*. Across the 12 journals our search identified 6,613 environmental health papers, of which 144 were identified in PubMed as RCTs. We reviewed these abstracts and removed human exposure experiments, trials of medical treatments (e.g. chelation therapy), and dietary supplements (e.g. calcium to reduce lead absorption). This left 41 RCTs focused on an environmental (or occupational) intervention [1-41]. The randomized intervention trials published to date have focused primarily on allergens [1-10], drinking water [11-15], household air pollution (HAP) from solid cooking fuels [16-20], lead [21-25], environmental tobacco smoke [26-30], and pesticides [31,32]. We did not exclude multiple publications from the same intervention study (e.g., the RESPIRE study [16-20]), so we may be overestimating of the role of RCTs in environmental health research.

***Search Terms***

Search (((((((((((("British medical journal"[Journal]) OR "JAMA : the journal of the American Medical Association"[Journal]) OR "The New England journal of medicine"[Journal]) OR "Lancet"[Journal]) OR "Pediatrics"[Journal]) OR "PLoS medicine"[Journal])) AND (((((((("journal article"[Publication Type]) NOT "case reports"[Publication Type]) NOT "editorial"[Publication Type]) NOT "guideline"[Publication Type]) NOT "letter"[Publication Type]) NOT "meta analysis"[Publication Type]) NOT "review"[Publication Type]) NOT ("review" OR "pooled analysis" OR "meta analysis" OR "meta-analysis" OR "meta analysis"[Title]))) AND ("2000"[Date - Publication] : "3000"[Date - Publication])) AND (("environmental health" OR "environmental pollution" OR "environmental pollutant" OR "environmental pollutants" OR "environmental chemical" OR "environmental chemicals" OR "environmental toxin" OR "environmental toxins" OR "environmental toxicant" OR "environmental toxicants" OR "environmental contaminant" OR "environmental contaminants" OR "air pollution" OR "polluted air" OR "water pollution" OR "polluted water" OR "contaminated water" OR "water contamination" OR "soil pollution" OR "polluted soil" OR "contaminated soil" OR "soil contamination" OR "particulate matter" OR "PM2.5" OR "PM10" OR "ozone" OR "nitrogen dioxide" OR "NO2" OR "NOx" OR "SO2" OR "solid fuel" OR "cookstoves" OR "cook stoves" OR "cooking stoves" OR "woodsmoke" OR "wood smoke" OR "radon" OR "asbestos" OR "environmental tobacco smoke" OR "secondhand smoke" OR "second hand smoke" OR "bisphenol A" OR "phthalate" OR "phthalates" OR ((("allergen" OR "allergens") OR ("asthma" and trigger*)) AND ("exposed" OR "exposure")) OR "endotoxin" OR "drinking water" OR "water disinfection" OR "arsenic" OR ("noise" AND (traffic* OR aircraft* OR airport* OR rail*)) OR "blood lead level" OR "blood lead levels" OR "blood lead concentration" OR "blood lead concentrations" OR "lead exposure" OR "lead exposures" OR "lead concentration" OR "lead concentrations" OR "lead poisoning" OR "Pb" OR "lead dust" OR "lead in dust" OR "cadmium" OR "chromium" OR "mercury exposure" OR "mercury exposures" OR "mercury level" OR "mercury levels" OR "mercury concentration" OR "mercury concentrations" OR "methylmercury" OR "methyl mercury" OR "heavy metals" OR (("pesticide" OR "pesticides" OR "DDT" OR "insecticide" OR "insecticides" OR "herbicide" OR "herbicides") NOT ("malaria" OR "bed net" OR "bednet" OR "lice")) OR "polychlorinated biphenyls" OR "polycyclic aromatic hydrocarbons" OR "dioxin" OR "dioxins" OR "benzene" OR "persistent organic" OR "endocrine disrupting" OR "endocrine-disrupting" OR "wastewater" OR "waste water" OR "climate change" OR "global warming" OR "heat wave" OR "heat waves" OR "heat-related" OR "heat related" OR "electromagnetic field" OR "electromagnetic fields"[Title/Abstract]))) NOT ((("warfarin" OR "coumadin" OR "heparin" OR anticoagul*[Title/Abstract]))))) AND (("randomized controlled trial"[Publication Type]) OR ("randomized controlled" OR "randomized intervention"[Title/Abstract])) Filters: Abstract available Sort by: Relevance

**References**

1. Custovic A, Simpson BM, Simpson A, Kissen P, Woodcock A, et al. (2001) Effect of environmental manipulation in pregnancy and early life on respiratory symptoms and atopy during first year of life: a randomised trial. Lancet 358: 188-193.

2. Woodcock A, Forster L, Matthews E, Martin J, Letley L, et al. (2003) Control of exposure to mite allergen and allergen-impermeable bed covers for adults with asthma. New England Journal of Medicine 349: 225-236.

3. Terreehorst I, Hak E, Oosting AJ, Tempels-Pavlica Z, de Monchy JGR, et al. (2003) Evaluation of impermeable covers for bedding in patients with allergic rhinitis. New England Journal of Medicine 349: 237-246.

4. Morgan WJ, Crain EF, Gruchalla RS, O'Connor GT, Kattan M, et al. (2004) Results of a home-based environmental intervention among urban children with asthma. New England Journal Of Medicine 351: 1068-1080.

5. Krieger J, Takaro TK, Allen C, Song L, Weaver M, et al. (2002) The Seattle-King County Healthy Homes Project: Implementation of a comprehensive approach to improving indoor environmental quality for low-income children with asthma. Environmental Health Perspectives 110: 311-322.

6. Takaro TK, Krieger JW, Song L (2004) Effect of environmental interventions to reduce exposure to asthma triggers in homes of low-income children in Seattle. Journal of Exposure Analysis and Environmental Epidemiology 14: S133-S143.

7. Swartz LJ, Callahan KA, Butz AM, Rand CS, Kanchanaraksa S, et al. (2004) Methods and issues in conducting a community-based environmental randomized trial. Environmental Research 95: 156-165.

8. Kercsmar CM, Dearborn DG, Schluchter M, Xue L, Kirchner HL, et al. (2006) Reduction in asthma morbidity in children as a result of home remediation aimed at moisture sources. Environmental Health Perspectives 114: 1574-1580.

9. Adgate JL, Ramachandran G, Cho SJ, Ryan AD, Grengs J (2008) Allergen levels in inner city homes: baseline concentrations and evaluation of intervention effectiveness. Journal of Exposure Science and Environmental Epidemiology 18: 430-440.

10. Noonan CW, Ward TJ (2012) Asthma randomized trial of indoor wood smoke (ARTIS): Rationale and methods. Contemporary Clinical Trials 33: 1080-1087.

11. Mausezahl D, Christen A, Pacheco GD, Tellez FA, Iriarte M, et al. (2009) Solar Drinking Water Disinfection (SODIS) to Reduce Childhood Diarrhoea in Rural Bolivia: A Cluster-Randomized, Controlled Trial. Plos Medicine 6.

12. Boisson S, Stevenson M, Shapiro L, Kumar V, Singh LP, et al. (2013) Effect of Household-Based Drinking Water Chlorination on Diarrhoea among Children under Five in Orissa, India: A Double-Blind Randomised Placebo-Controlled Trial. Plos Medicine 10.

13. Hellard ME, Sinclair MI, Forbes AB, Fairley CK (2001) A randomized, blinded, controlled trial investigating the gastrointestinal health effects of drinking water quality. Environmental Health Perspectives 109: 773-778.

14. Eisenberg JNS, Hubbard A, Wade TJ, Sylvester MD, LeChevallier MW, et al. (2006) Inferences drawn from a risk assessment compared directly with a randomized trial of a home drinking water intervention. Environmental Health Perspectives 114: 1199-1204.

15. Hartinger SM, Lanata CF, Hattendorf J, Gil AI, Verastegui H, et al. (2011) A community randomised controlled trial evaluating a home-based environmental intervention package of improved stoves, solar water disinfection and kitchen sinks in rural Peru: Rationale, trial design and baseline findings. Contemporary Clinical Trials 32: 864-873.

16. Smith KR, McCracken JP, Weber MW, Hubbard A, Jenny A, et al. (2011) Effect of reduction in household air pollution on childhood pneumonia in Guatemala (RESPIRE): a randomised controlled trial. Lancet 378: 1717-1726.

17. McCracken JP, Smith KR, Diaz A, Mittleman MA, Schwartz J (2007) Chimney stove intervention to reduce long-term wood smoke exposure lowers blood pressure among Guatemalan women. Environmental Health Perspectives 115: 996-1001.

18. Smith KR, McCracken JP, Thompson L, Edwards R, Shields KN, et al. (2010) Personal child and mother carbon monoxide exposures and kitchen levels: Methods and results from a randomized trial of woodfired chimney cookstoves in Guatemala (RESPIRE). Journal of Exposure Science and Environmental Epidemiology 20: 406-416.

19. Thompson LM, Bruce N, Eskenazi B, Diaz A, Pope D, et al. (2011) Impact of Reduced Maternal Exposures to Wood Smoke from an Introduced Chimney Stove on Newborn Birth Weight in Rural Guatemala. Environmental Health Perspectives 119: 1489-1494.

20. McCracken J, Smith KR, Stone P, Diaz A, Arana B, et al. (2011) Intervention to Lower Household Wood Smoke Exposure in Guatemala Reduces ST-Segment Depression on Electrocardiograms. Environmental Health Perspectives 119: 1562-1568.

21. Lanphear BP, Eberly S, Howard CR (2000) Long-term effect of dust control on blood lead concentrations. Pediatrics 106.

22. Brown MJ, McLaine P, Dixon S, Simon P (2006) A randomized, community-based trial of home visiting to reduce blood lead levels in children. Pediatrics 117: 147-153.

23. Jordan CM, Yust BL, Robison LL, Hannan P, Deinard AS (2003) A randomized trial of education to prevent lead burden in children at high risk for lead exposure: Efficacy as measured by blood lead monitoring. Environmental Health Perspectives 111: 1947-1951.

24. Binns HJ, Gray KA, Chen TY, Finster ME, Peneff N, et al. (2004) Evaluation of landscape coverings to reduce soil lead hazards in urban residential yards: The Safer Yards Project. Environmental Research 96: 127-138.

25. Yiin LM, Lu SE, Sannoh S, Lim BS, Rhoads GG (2004) Evaluation of cleaning methods applied in home environments after renovation and remodeling activities. Environmental Research 96: 156-162.

26. Hovell MF, Meltzer SB, Wahlgren DR, Matt GE, Hofstetter R, et al. (2002) Asthma management and environmental tobacco smoke exposure reduction in Latino children: A controlled trial. Pediatrics 110: 946-956.

27. El-Mohandes AAE, Kiely M, Blake SM, Gantz MG, El-Khorazaty MN (2010) An Intervention to Reduce Environmental Tobacco Smoke Exposure Improves Pregnancy Outcomes. Pediatrics 125: 721-728.

28. Lanphear BP, Hornung RW, Khoury J, Yolton K, Lierl M, et al. (2011) Effects of HEPA Air Cleaners on Unscheduled Asthma Visits and Asthma Symptoms for Children Exposed to Secondhand Tobacco Smoke. Pediatrics 127: 93-101.

29. Harutyunyan A, Movsisyan N, Petrosyan V, Petrosyan D, Stillman F (2013) Reducing Children's Exposure to Secondhand Smoke at Home: A Randomized Trial. Pediatrics 132: 1071-1080.

30. Stotts AL, Northrup TF, Schmitz JM, Green C, Tyson J, et al. (2013) Baby's Breath II protocol development and design: A secondhand smoke exposure prevention program targeting infants discharged from a neonatal intensive care unit. Contemporary Clinical Trials 35: 97-105.

31. Thompson B, Coronado GD, Vigoren EM, Griffith WC, Fenske RA, et al. (2008) Para Ninos Saludables: A community intervention trial to reduce organophosphate pesticide exposure in children of farmworkers. Environmental Health Perspectives 116: 687-694.

32. Bradman A, Salvatore AL, Boeniger M, Castorina R, Snyder J, et al. (2009) Community-based intervention to reduce pesticide exposure to farmworkers and potential take-home exposure to their families. Journal of Exposure Science and Environmental Epidemiology 19: 79-89.

33. Mendell MJ, Fisk WJ, Petersen MR, Hines CJ, Dong M, et al. (2002) Indoor particles and symptoms among office workers: Results from a double-blind cross-over study. Epidemiology 13: 296-304.

34. Skulberg KR, Skyberg K, Kruse K, Eduard W, Djupesland P, et al. (2004) The effect of cleaning on dust and the health of office workers - An intervention study. Epidemiology 15: 71-78.

35. Emerson PM, Lindsay SW, Alexander N, Bah M, Dibba SM, et al. (2004) Role of flies and provision of latrines in trachoma control: cluster-randomised controlled trial. Lancet 363: 1093-1098.

36. Luby SP, Agboatwalla M, Feikin DR, Painter J, Billhimer W, et al. (2005) Effect of handwashing on child health: a randomised controlled trial. Lancet 366: 225-233.

37. Gerr F, Marcus M, Monteilh C, Hannan L, Ortiz D, et al. (2005) A randomised controlled trial of postural interventions for prevention of musculoskeletal symptoms among computer users. Occupational and Environmental Medicine 62: 478-487.

38. McCreanor J, Cullinan P, Nieuwenhuijsen MJ, Stewart-Evans J, Malliarou E, et al. (2007) Respiratory effects of exposure to diesel traffic in persons with asthma. New England Journal of Medicine 357: 2348-2358.

39. Marks GB, Ezz W, Aust N, Toelle BG, Wei XA, et al. (2010) Respiratory Health Effects of Exposure to Low-NOx Unflued Gas Heaters in the Classroom: A Double-Blind, Cluster-Randomized, Crossover Study. Environmental Health Perspectives 118: 1476-1482.

40. Langrish JP, Li X, Wang SF, Lee MMV, Barnes GD, et al. (2012) Reducing Personal Exposure to Particulate Air Pollution Improves Cardiovascular Health in Patients with Coronary Heart Disease. Environmental Health Perspectives 120: 367-372.

41. Sathyanarayana S, Alcedo G, Saelens BE, Zhou C, Dills RL, et al. (2013) Unexpected results in a randomized dietary trial to reduce phthalate and bisphenol A exposures. Journal of Exposure Science and Environmental Epidemiology 23: 378-384.
